# Frequency of seizure attack and associated factors among patients with epilepsy at University of Gondar Referral Hospital: a cross-sectional study, Gondar, North West Ethiopia, 2017

**DOI:** 10.1186/s13104-018-3761-3

**Published:** 2018-09-06

**Authors:** Mekdes Tigistu, Telake Azale, Habtamu Kebebe, Temesgen Yihunie

**Affiliations:** 1 Institute of Health Science, Wollega University, P.O. Box 395, Nekemte, Ethiopia; 2 Institute of Public Health, College of Medical and Health Sciences, University of Gondar, P.O. Box 196, Gondar, Ethiopia

**Keywords:** Seizure, Frequency, Epileptic patient, Gondar, Zero-Inflated Negative Binomial

## Abstract

**Objective:**

About three-fourth of adults with new-onset epilepsy become seizure-free with current anti-epileptic drugs, but around one-fourth of the patients continue to experience seizure which increases the risk of accident, disability, death and treatment side effects. Therefore, this study aimed to address the gap in determining the magnitude of the number of seizure attacks and identify the factors that provoke a repeated seizure in a patient with epilepsy.

**Results:**

A total of 166(40.68%) study participants were experienced seizure attacks with a minimum of one and a maximum of seventeen times attacks. Perceived exposure to noise (adjusted incidence risk ratio (AIRR) = 1.91, 95% confidence interval (CI) [1.46, 2.49]), light (AIRR = 1.48, 95% CI [1.09, 2.00]), head injury (AIRR = 1.71, 95% CI [1.14, 2.57]) and sleep deprivations (AIRR = 1.41, 95% CI [1.02, 1.94]) were associated with increased incidence of seizure, while adherence adjusted odds ratio (AOR) = 18.18, 95% CI [3.49, 94.63]), being in middle wealth index (AOR = 3.52, 95% CI [1.14, 11.02]) and being in rich wealth index (AOR = 4.05, 95% CI [1.54, 10.69]) were associated with inflation of zero count.

## Introduction

Epilepsy is one of the common chronic neurologic disorders characterized by recurrent seizure that is a brief period of uncontrolled involuntary shaking. Patient with epilepsy may continue to experience any types of seizure while getting antiepileptic drugs (AEDs) treatment [1, 2]. In 2008, globally there were 50 million patients with epilepsy. Form these 40 million were from developing countries, and among those patients, 90% of them do not receive appropriate treatment for their problem. In 2010 more than 62 million epileptic people live in low and middle-income countries [3, 4].

Patients with epilepsy have poor health outcomes, including greater psychological distress, depression, anxiety, employment restriction, more physical injuries such as fractures and burns, and increased mortality, besides epileptic seizures result in devastating social consequences which result in poor quality of life [1]. When the number of seizure frequency increases, depression and perceived stigma also increase which further results in the devastating social consequences and complication of patients’ quality of life [5].

A study from Ethiopia found about 58% of the patients who developed generalized tonic–clonic seizure at baseline evaluation with the frequency of ≤ 8 times, 23.2% of them died [6]. Another study also showed that 20 of the 316 persons with epilepsy (6.3%) died over a 2-year period, most deaths occurred due to status epilepticus and burn [7].

The Federal Ministry of Health in Ethiopia has recently issued a Mental Health Strategy that that having an objective providing quality mental health services [8]. The ultimate goal of treating the epileptic patients is to maintain seizure-free state but in spite of anti-epileptic treatment, some patients may continue to experience seizure. Therefore, identifying seizure-provoking factors among patients who are on AED treatment is very crucial to improve the patients’ quality of life.

The aim of this study was to determine the frequency of seizure as well as to identify important seizure-provoking factor so this study tried to address the research gap in this specific research area.

## Main text

### Methodology

An institution-based cross-sectional study was conducted from March to May 2017 in University of Gondar Referral Hospital which is located in North Gondar administrative zone, Amhara National Regional State, which is located 750 km Northwest of Addis Ababa. According to 2016 Federal Democratic Republic of Ethiopia Central Statistical Agency, the total population of Gondar town estimated to be 341,991 [9]. Currently, Gondar town has one Referral Hospital (UOGRH), which have 1000 beds that believed to serve for over five million people in Gondar town and its environs. It has been serving the community since 1954. The hospital has more than 700 health professionals. The average number of patients with epilepsy that has followed in UOGRH per month is 396.

The sample size of this study was determined by using modified Signorini [10] proposed methods of normalized Poisson. Based on the above simulation approach the calculated sample size of this study was estimated to be 389 with 95% CI and 90% power [11]. The final sample size including 5% non-response rate became 408 epileptic patients. Systematic random sampling employed to select the eligible study participants. Patients with epilepsy aged above 15 years and who have been taking antiepileptic treatment at least for the last 2 months was included while there was no patients excluded from the study.

Trained seven B.Sc. nurses collected data through face-to-face interview and medical chart review. The questionnaire contains socio-demographic and economic factors, behavioral factors, clinical factors, environmental factors, treatment-related factors, and seizure-related factors, four MMAS and wealth index questions.

Structured questionnaire were prepared in English and translated into Amharic then, to check the consistency of the tool the Amharic version translated into English. The questionnaire was carefully evaluated and pre-tested on 5% of the sample size which became 21 patients with epilepsy at Tseda Health Center prior to the actual data collection. In addition to the principal investigator, two supervisors were responsible for monitoring the data collection process. Before data entry, the collected data carefully examined for completeness by the principal investigator.

The collected data entered into Epi-info version 7 and cleaned, analyzed using STATA version 14. Descriptive and summary statistics were carried out and presented using graphs and tables. Both bi-variable and multivariable analysis performed to determine the association between seizure frequency and explanatory variables using zero-inflated negative binomial regression model because there was over dispersions and significant zero count. Variables with a P-value < 0.2 in the bi-variable analysis were selected for multivariable analysis. Finally, 95% CI of AIRR and AOR were presented and interpreted accordingly.

### Results

A total of 408 respondents were interviewed and the overall response rate of the respondents was 100%. The mean age of the respondents was 29 years with standard deviation (SD) of 13.8 and majority of the respondents 175(42.9%) were between 15 and 24 years. Around 243(59.5%) of the respondents are male and majority 247(60.5%) of them were from an urban residence. The marital status of respondents’ shows about 233(57.1%) of them were single. Only 53(13%) of the respondents have an educational status of college and university. Regarding their occupation around 133(32.6%) of the respondents are students and about a quarter 109(26.72%) of the respondents were in a higher economic level.

The common anti-epileptic drug used to treat epilepsy is Phenobarbital and from the current medication of the respondents, Phenobarbital reported by 253 respondents, which accounts for 62.5% of current treatment and mono treatment modality accounts around 75.5%. Anti-epileptic treatment is affordable for 63.5% of the respondents, but from the 408 study participants, 9.31% of the respondents complained about treatment-related side effects. Based on MMAS, 24.57% of the respondents had low adherence and 44.23% of them had high adherence for an anti-epileptic drug (see Table 1).

**Table 1:** Socio-demographic, economic and treatment related characteristics of the study participants of seizure frequency and associated factors among patient with epilepsy in University of Gondar Referral Hospital, 2017 (n = 408)

Variables with category	Frequency	Percentage (%)
Age		
15–24	175	42.89
25–34	123	30.15
35–44	49	12.01
45–54	24	5.88
55–64	25	6.13
65–80	12	2.94
Sex		
Male	243	59.56
Female	165	40.44
Residence		
Urban	247	60.54
Rural	161	39.46
Marital status		
Single	233	57.11
Married	138	33.82
Divorced	26	6.37
Widowed	11	2.7
Educational level		
No formal education	158	38.72
Primary	118	28.91
Secondary	79	19.36
Higher	53	13
Occupation		
Student	133	32.6
Farmer	111	27.21
Merchant	33	8.09
Employee	45	11.03
Housewife	50	12.25
Other	36	8.82
Wealth index		
Poor	153	37.5
Middle	146	35.78
Rich	109	26.72
Type of drug when treatment started		
Phenobarbital	255	62.5
Phenytoin	55	13.48
Carbamazepine	15	3.68
Two or more drugs	79	19.36
Other^a^	4	0.98
Adherence		
Low	100	24.57
Moderate	127	31.2
High	180	44.23
Duration of treatment (years)		
< 1	52	12.75
1–5	204	50
5–10	98	24.02
> 10	54	13.23
Treatment modality		
Mono	308	75.49
Poly	100	24.51
Current medication		
Phenobarbital	253	62.01
Phenytoin	31	7.6
Carbamazepine	13	3.19
Two or more drugs	106	25.98
Other^a^	5	1.22
Tapering in the last 2 months		
Yes	21	5.15
No	387	94.85
Treatment side effect		
Yes	38	9.31
No	370	90.69
Treatment affordability		
Yes	259	63.48
No	149	36.52

^**a**^Other: lamotrigine, sodium valproate and haloperidol

We assessed the patients’ emotional responses to self-perceived stressors. Anxiety and anger holds the first and the second place, which accounts 14.95% and 10.29% respectively. Respondent’s perception regarding environmental factors and seizure attack asked and about 4.6% of them reported as humidity provoke seizure attack. Around 21.5%, 38.7% and 9.07% of the respondents reported to light, noise and weather change respectively. Concerning the experience of depression, about 154(37.7%) patents have developed depression.

**Fig. 1:**
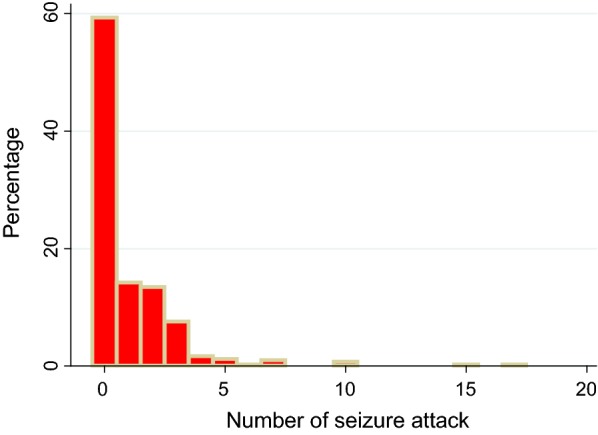
Number of seizure attack in the last 2 months among study participants of seizure frequency and associated factors among patients with epilepsy in University of Gondar Referral Hospital, 2017 (n = 408)

The minimum and maximum numbers of seizure attacks in the last 2 months were 1 and 17 respectively. Two hundred forty-two (59.3%) of the respondents had no experienced seizure attack and only one respondent reported 17 seizure attacks. The most repeatedly reported numbers of seizure attack were 1, 2 and 3 with which reported by 58(14.2%), 55(13.4%) and 31(7.6%) respondents respectively (see Fig. 1).

In multivariable analysis, exposure to noise (AIRR = 1.91, 95% CI [1.46, 2.49]), light (AIRR = 1.48, 95% CI [1.09, 2.00]), head injury (AIRR = 1.71, 95% CI [1.14, 2.57]) and sleep deprivations (AIRR = 1.41, 95% CI [1.02, 1.94]) were associated with increased incidence of seizure. Adherence (AOR = 18.18, 95% CI [3.49, 94.63]), being in middle wealth index (AOR = 3.52, 95% CI [1.14, 11.02]), being in rich wealth index (AOR = 4.05, 95% CI [1.54, 10.69]) and sleeping hours (AOR = 1.04, 95% CI [1.27, 1.40]) were associated with inflation of zero count (see Table 2).

**Table 2:** Risk factors of seizure frequency in the last 2 months among patients with epilepsy in University of Gondar Referral Hospital from Zero Inflated Negative Binomial Poisson Regression model, 2017 (n = 408)

Variables	Seizure frequency			CIRR(95% CI)	AIRR(95% CI)	P-value
	0	1_5	≥ 6			
Noise						
No	169	80	1	1	1	< 0.001
Yes	73	76	9	2.89(2.08, 4.01)	*1.91*(*1.46*, *2.49*)***	
Light						
No	208	108	4	1	1	0.011
Yes	34	48	6	2.33(1.59, 3.41)	*1.48*(*1.09*, *2.00*)*	
Humidity						
No	234	147	8	1	1	0.147
Yes	8	9	2	2.46(1.18, 5.13)	1.62(0.84, 3.13)	
Head injury						
No	238	155	8	1	1	0.009
yes	3	12	2	3.50(1.67, 7.34)	*1.71*(*1.14*, *2.57*)**	
Meal on time						
No	203	188	6	1	1	0.192
Yes	39	38	4	1.69(1.12, 2.55)	1.21(0.90, 1.62)	
Sleep deprivation						
No	216	213	6	1	1	0.035
Yes	26	43	4	2.21(1.48, 3.36)	*1.41*(*1.02*, *1.94*)*	
Strenuous exercise						
No	222	133	6	1	1	0.607
Yes	20	23	4	1.80(1.09, 2.99)	1.10(0.76, 1.60)	
Side effect						
No	222	142	6	1	1	0.316
Yes	20	14	4	1.87(1.08, 3.24)	1.33(0.76, 2.35)	

Variables	Seizure		COR(95% CI)	AOR(95% CI)	P-value
	Zero	Non-Zero			
Inflation model					
Adherence					
Non adherent	95	132	1	1	
Adherent	146	34	0.17(0.11, 0.26)	*18.18*(*3.49*, *94.63*)***	0.001
Wealth index					
Poor	70	83	1	1	
Middle	100	46	0.38(0.24, 0.62)	*3.52*(*1.14*, *11.02*)*	0.029
Rich	72	37	0.43(0.26, 0.72)	*4.05*(*1.54*, *10.69*)**	0.005
A Unit raise in sleeping hours			0.89(0.81, 0.98)	1.04(0.78, 1.40)	0.750

Italics show significance

*** P-value less than 0.001, ** P-value less than 0.01, * P-Value less than 0.05, 1-Reference Category

### Discussion

The number of seizure attack in the last 2 months was reported by 166(40.7%) of the participants with a minimum of one and a maximum of 17 attacks. The finding of this study were lower than another study, which found ten or more seizures in the last month prior to the interview in over 50% of the study participants [12]. This may be due to a small sample size used by the other study.

The respondents who reported sleep deprivation in the last 2 months had a 41% times (AIRR = 1.41, 95% CI [1.02, 1.94]) higher incidence rate of seizure attack as compared to their counterparts. Studies from Denmark and Norway also support this finding. Patients with generalized epilepsy were found to be highly sensitive to sleep deprivation and seizure attack [13, 14]. The possible reason could be a majority of respondents in the present study diagnosed by epilepsy with generalized seizure type. The baseline assessment of a controlled prospective study also found higher seizure frequency in sleep-deprived group. In another study, which evaluated the perceived triggering factors, sleep deprivation was reported as one of the triggering factors [15]. However, the same study showed no statistically significant association between sleep deprivation and seizure attack during follow up [16]. This may be because the study was limited to patients with refractory epilepsy and the participants did not consume any illicit drug. However, in the present study, there were respondents who consume alcohol, cigarette and chat.

The incidence of seizure attack among individuals reported to have head injury was 14% (AIRR = 1.71, 95% CI [1.14, 2.57]) than their counterparts. It had been reported as having head injury will increase the risk of developing seizure attack, which could be or not an epileptic seizure. But there is an evidence suggesting occurrences of epilepsy after head injury [17]. So, the entire risk of having seizure attack had increased among individuals reported to have head injury in this study.

Study participants who perceived light as a seizure precipitant had 48% higher risk of (AIRR = 1.48, 95% CI [1.09, 2.00]) experiencing repetitive seizure attacks than their counterpart. This finding is in line with study that, reported patients with generalized epilepsy were found to be highly sensitive to flickering light than those with localization-related epilepsy [13]. This consistency could be because the majority of the patients with epilepsy in this study were diagnosed by generalized seizure type. The incidence rate of seizure attack is 90% (AIRR = 1.91, 95% CI [1.46, 2.49]) higher among respondents who reported noise as a seizure precipitant factor as compared to those who do not report.

This study also found respondents who were adherent to their AED treatment had a lower risk of seizure attack which enhances the odds of zero value for seizure frequency as compared to non-adherent. This is in line with a retrospective study and a cross-sectional studies which found a statistically significant association between the higher risk of seizure and non-adherence to AED [18, 19]. A pilot survey on the relationship between poor medication adherence and seizures also found a statistically significant association between missing dose of a medication and higher risk of seizure attack [20].

The odds of seizure frequency to be zero was three times (AOR = 3.52, 95% CI [1.14, 11.02]) among individuals in middle economic status and four times (AOR = 4.05, 95% CI [1.54, 10.69]) among rich individuals as compared to low economic status. This finding also supported by different literatures which reports aggravation of epilepsy in poor countries and those low-income countries [21, 22] Individuals having better-leaving condition economically could afford and access anti-epileptic drugs than those in low economic status. Once they afford the drugs, the probability of adhering to drug intake will be enhanced. Therefore, this fact could not only minimize the frequency of seizure attack, but also its occurrences totally.

### Conclusions and recommendations

The seizure frequency in this study found to be higher than the expected seizure frequency, which is preferably zero. The perceived exposure to noise and flickering light, having a head injury and experiencing sleep deprivation associated with increased incidence of seizure attack with a higher order of frequency, while being adherent to AID and better wealth index (middle and rich) prevent the frequency of seizure attacks.

Care providers of epileptic patients need to consider options to control seizure attacks by creating a conducive environment by minimizing the exposure of epileptic patients to unpleasant noise, flecking light. Prevention of sleep deprivation and head injury to epileptic patients are significant actions to prevent the increased incidence of seizure attacks. For effective control of seizure, it would be better if a health-professionals, community and family members of patient with epileptic seizures put a great effort on enhancing adherence to AED. We recommend studies incorporating multi-center on this topic for the better estimate of the association as well as the temporal relationship of different determinant factors and seizure.

## Limitations of the study

Cross-sectional nature of the study and inability to generalize the findings of this study to patients with epilepsy outside the study area are the limitations of this study. This problem needs to be addressed by further multi-centered prospective studies.

## Abbreviations

AED: anti-epileptic drug
AOR: adjusted odds ratios
AIRR: adjusted incidence rate ratio
AIC: Akaike information criteria
CI: confidence interval
COR: crudes odds ratios
BIC: Bayesian information criteria
IRR: incidence rate ratio
MMAS: Morisky Medication Adherence Scale
UOGRH: University of Gondar Referral Hospital
ZINB: Zero-Inflated Negative Binomial
